# The Study of a New Modified Bicanalicular Intubation for the Repairment of Traumatic Canalicular Laceration

**DOI:** 10.1155/2019/8435185

**Published:** 2019-01-27

**Authors:** Miaomiao Zhang, Bin Li, Ning Zhang

**Affiliations:** Department of Ophthalmology, The Second People's Hospital of Jinan, 148# Jingyi Road, Jinan 250001, China

## Abstract

**Introduction:**

To investigate the efficacy and safety of a modified bicanalicular intubation (MBCI) used in canalicular laceration.

**Materials and Methods:**

This study is a retrospective consecutive chart review. A total of 43 eyes from 43 patients (36 males and 7 females) who underwent canalicular intubation were enrolled. Success rate was determined at 6 months after the surgery. Anatomical success was determined by diagnostic probing and irrigation; functional success was determined by asking patients about tearing.

**Results:**

Irrigation of the lacrimal passages in all 43 eyes showed that they were free from obstruction. The anatomical success was 100%, and 37 eyes (86%) achieved functional success. 6 eyes (14%) could not achieve functional success because there were some residual symptoms under irritating conditions, such as wind or winter weather, among which 2 eyes had bicanalicular lacerations and 4 eyes had lower canalicular laceration before surgery. There were no other complications observed in this study.

**Conclusions:**

The MBCI was simple and safe for using in canalicular laceration.

## 1. Introduction

Canalicular laceration may occur at any age [1, 2], especially in children and young adults and commonly affects the inferior canaliculus [3]. In addition, both direct and indirect injuries at the medial canthal region may result in canalicular laceration [1, 4]. It is difficult to deal with when both superior and inferior canaliculi were lacerated [5–7]. Canalicular laceration frequently accompanies other ocular injuries, including eyelid and globe lacerations [8]. Unrepaired canalicular lacerations may cause inflammation, scar, canalicular stenosis, and obstruction, leading to subsequent epiphora [3]. Several methods are available for reconstructing the lacerated canaliculus, including repair of the lacerated eyelid without a lacrimal stent, intubation of the lacerated duct with monocanalicular or bicanalicular stents with or without mucosal anastomosis, or early canaliculodacryocystorhinostomy [1, 9–13].

Most surgeons believe that reconstruction of a lacerated canaliculus with a stent is necessary [9]. Both bicanalicular and monocanalicular intubation have been reported for reconstructing traumatic canalicular laceration [10, 11, 13, 14], but with no consensus with respect to which is the best technique.

Herein, according to the accumulation of our clinical work, we describe a modified bicanalicular intubation (MBCI) technique for the treatment of canalicular laceration.

## 2. Materials and Methods

**Figure 1:**
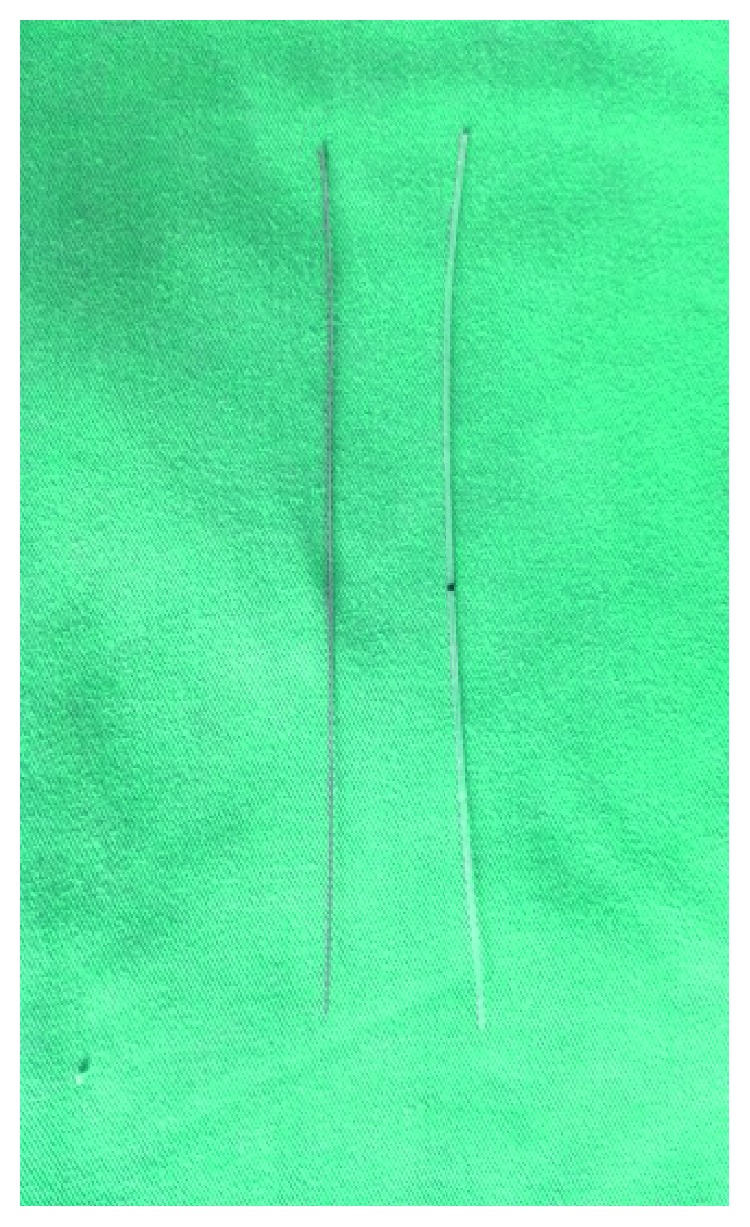


MBCI is a new bicanalicular silicone tube designed. This tube comprises 2 parts. The first part is a polyurethane elastomer tube with 2 blind tips (Figure 1). There is a black mark spot in the middle of the polyurethane elastomer tube for positioning when implanted. There are also some holes located on the side of the tube for drainage and metal probe insert. The second part is a metal probe that lies within the stent lumen and works as a guiding probe, which is similar to an arterial catheter, facilitates the insertion of the stent (Figure 1). Once the lacrimal canaliculus is intubated, the metal probe is withdrawn, and the polyurethane elastome stent remains in the canaliculus (Figure 1).

The main material of MBCI is polyurethane elastomer, which both with the toughness and rigidity has excellent biocompatibility [15]. The hardness is Shore 74D (the hardness of implanted silicone commonly used in the human body is Shore 30-50D).

### 2.1. Patients

This study is a retrospective consecutive chart review. A total of 43 eyes from 43 patients (36 males and 7 females) underwent canalicular intubation from October 2014 to June 2016 in the Second People's Hospital of Jinan. The protocol was approved by the Institutional Ethics Committee of the Second People's Hospital of Jinan and conformed to the tenets of the Declaration of Helsinki. The age ranged from 22 to 53 years (mean age 32.54 years). The duration from the time of canalicular injury to the surgery ranged from 1 h to 3 days. In patients with suspected canalicular laceration, presence of a laceration was confirmed by canalicular probing. Demographic and other data on the type of injury and concomitant eye and noneye injuries were collected for each patient. Among the 43 eyes, 32 (74.4%) had lower canalicular lacerations, 4 (9.3%) had upper canalicular lacerations, and 7 (16.3%) had bicanalicular lacerations. A consent form was obtained from each patient before the surgery.

### 2.2. Surgical Procedures

**Figure 2:**
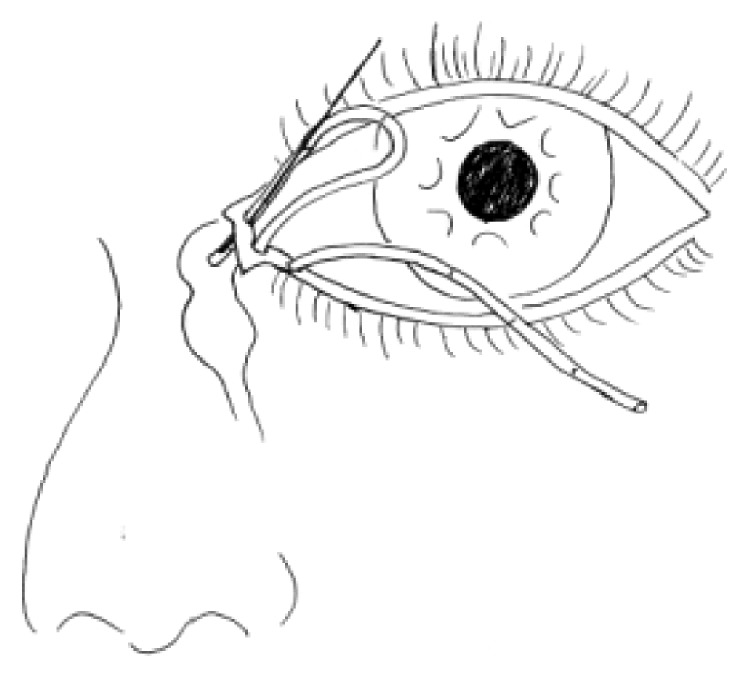


**Figure 3:**
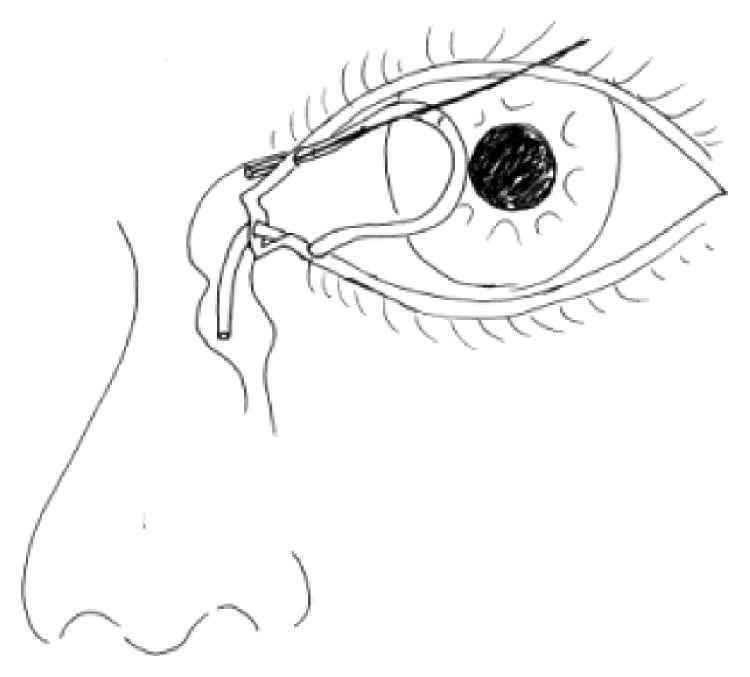


**Figure 4:**
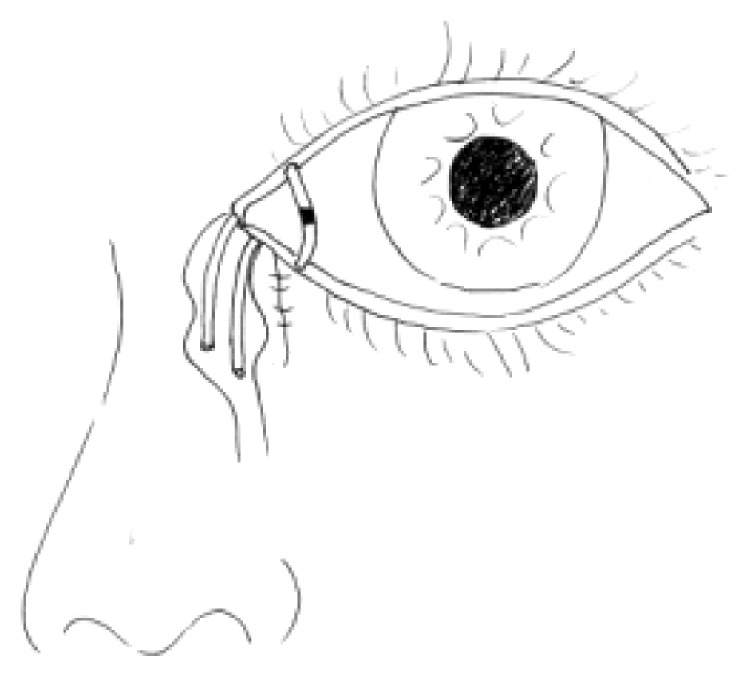


**Figure 5:**
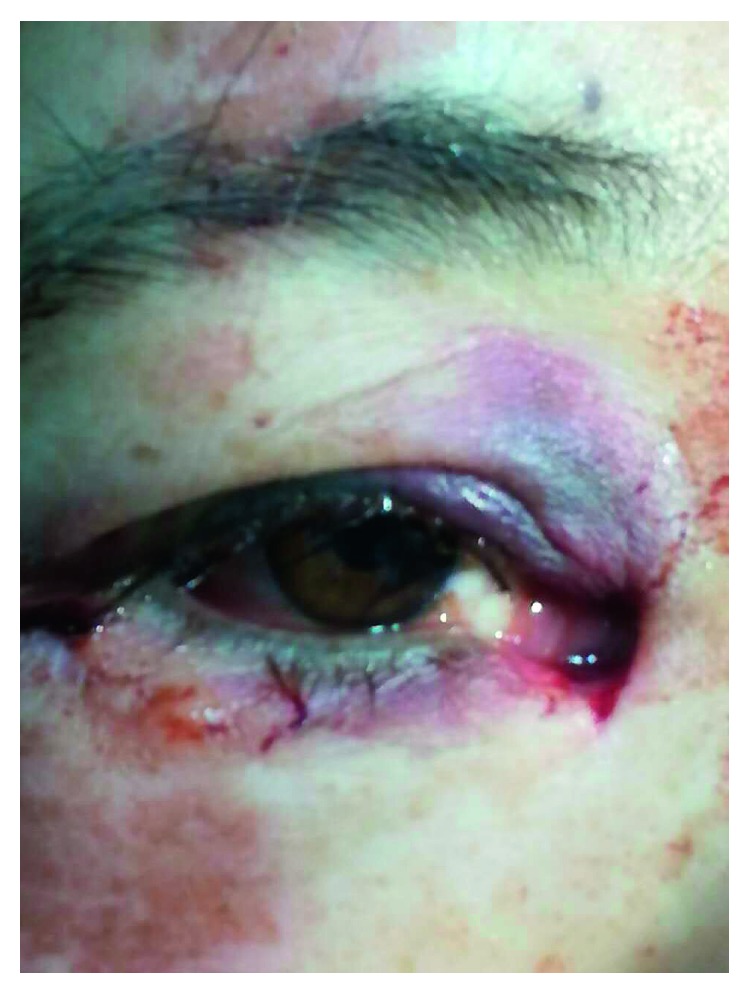


**Figure 6:**
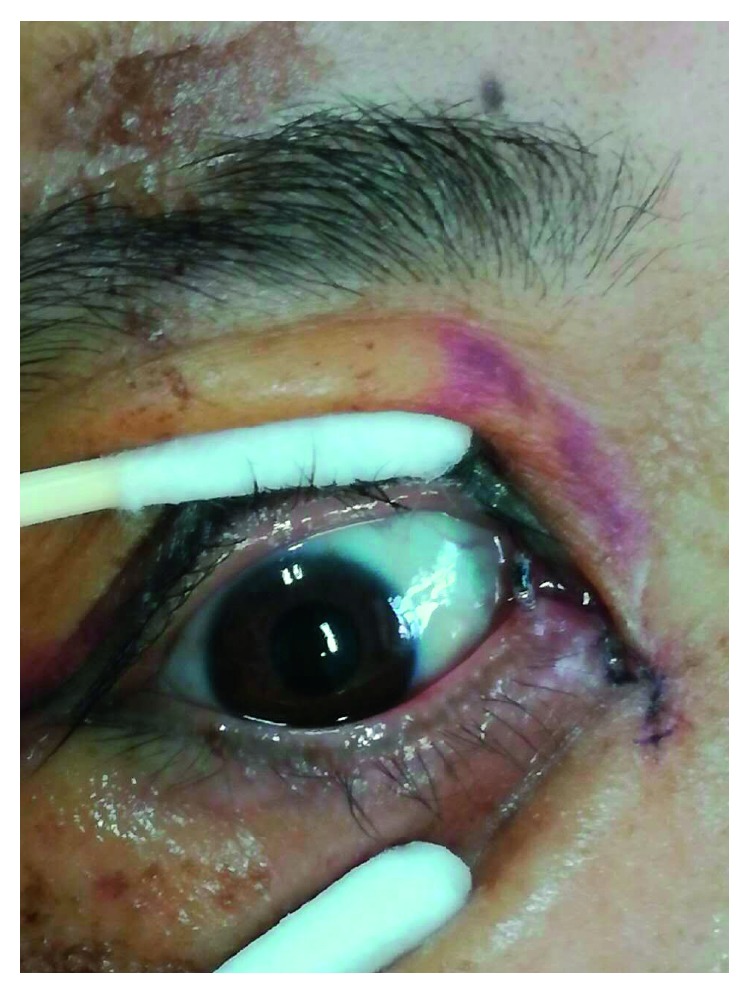


**Figure 7:**
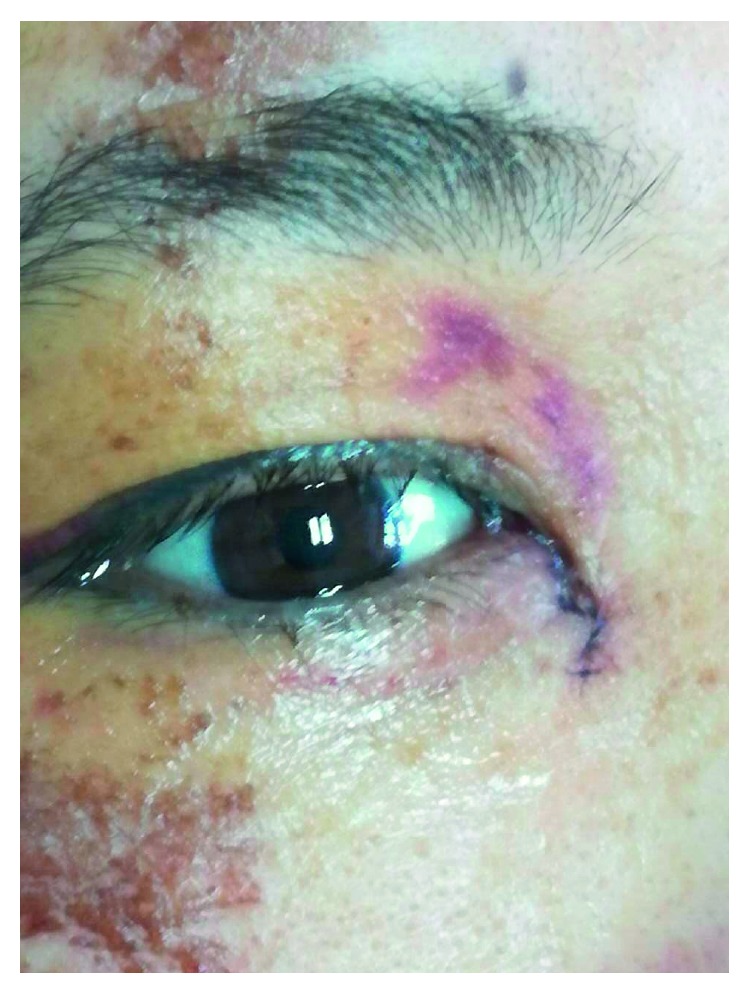


For all patients, the surgical procedures and diagnostic probing at follow-up visits were performed by the same surgeon. Canalicular laceration was repaired under a surgical microscope. The procedure was performed under local infiltration anesthesia with 2% lidocaine hydrochloride. First, the proximal portion of the canaliculus was explored. Subsequently, the inferior punctum was dilated using a punctum dilator and MBCI was then inserted in the punctum (Figure 2). Following intubation of the proximal and distal portions of the lacerated canaliculus, the tube was fixed in the punctum and the metal probe was withdrawn from the stent. Subsequently, the other end of the MBCI was inserted in superior puncta with leaving a loop of tubing extending between the inferior and superior puncta. After that, the 2 ends of the lacerated canaliculus were approximated using a 7-0 Vicryl suture. Eventually, the eyelid margin and other parts of the eyelids were repaired using 6-0 silk sutures (Figure 3). At last, the position of the MBCI was adjusted until the black mark spot was located in the inner canthus, without any suture fixation (Figure 4). After the surgery, chloramphenicol and betamethasone eye drops (4 times a day for 5 days) and tetracycline eye ointment (twice a day) were prescribed to all patients (Figures 5–7).

### 2.3. Removal of the Polyurethane Elastomer Tube

All subjects were asked to visit the hospital on the first and third days following the surgery, at the end of the first week after the surgery, and at the end of the first, third, and sixth month after the surgery.

The tube was removed at 3 months after the surgery, when the patients had experienced relief from tearing and irrigation (Figure 4). After a drop of topical ophthalmic anesthetic (0.4% Oxybuprocaine Hydrochloride Eye Drops, Santen Pharmaceutical Co. Ltd., Shiga Plant, Japan) was instilled into the conjunctival sac, the MBCI was pulled out with small, blunt forceps. Irrigation (with 0.3% Tobramycin Eye Drops (s.a. ALCON-COUVREUR n.v.)) was administered to the patients immediately after tube removal to clear the discharge in the lacrimal passage.

Success rate was determined at 6 months after the surgery. Anatomical success was determined by diagnostic probing and irrigation; functional success was determined by asking patients about tearing.

Statistical analysis was performed using SPSS version 17 (SPSS Inc., Chicago, IL).

## 3. Results

MBCI was performed successfully in all eyes without any intraoperative complications during the surgery. All the tubes were left in place for 3 months after the surgery. All the tubes were removed successfully in the outpatient department. The follow-up after tube removal ranged from 6 to 25 months (mean 11.7 ± 5.1 months).

Irrigation of the lacrimal passages in all 43 eyes showed that they were free from obstruction. The anatomical success was 100%, and 37 eyes (86%) achieved functional success. Six eyes (14%) could not achieve functional success because there were some residual symptoms under irritating conditions, such as wind or winter weather, among which, 2 eyes had bicanalicular lacerations and 4 eyes had lower canalicular laceration before surgery. There were no other complications observed in this study.

No intraoperative complication occurred. Spontaneous tube loss was not observed. Intracanalicular migration of the stent, chronic irritation, and formation of granulation tissue were not observed in any patient.

## 4. Discussion

Silicone is the most widely used tubing material to prevent canalicular obstruction. For the canalicular stenting, one essentially must choose from either a monocanalicular stenting, an annular stent assisted by pigtail probe, or BCI stents [9, 16–21]. Recently, it has been reported that monocanalicular stent extrusions may occur within 1 month and with a 11.1% extrusion rate, especially in upper canalicular lacerations, resulting in potential damage to the ocular surface [8]. Therefore, this technique is not suitable for use in patients with combined upper and lower canalicular lacerations [22]. Kersten and Kulwin [23] reported “one-stitch” canalicular repair, a simplified approach for repair canalicular lacerations. But there is still probability of inflammation granuloma caused by remained suture near the canaliculus, which theoretically could lead to a bigger scar in the anastomosis site, and could also possibly lead to lacrimal canalicular obstruction.

BCI, which was developed by Crawford [24] and Guibor [25], involves the passage of the tube through the inferior and superior puncta, leaving a loop of tubing extending between the inferior and superior puncta. The approach has been widely adopted by many ophthalmologists since the 1970s because of its simplicity, safety, efficacy, and minimal invasiveness [26]. However, the traditional BCI stent has some disadvantages, including the potential damage to the normal nasolacrimal duct, the Hasner valve, and the ocular surface. To avoid the drawbacks mentioned above, a number of developments have been reported, such as the use of pigtail [9, 21, 27], which some surgeons find challenging to master, especially to the bicanalicular lacerations.

To overcome the disadvantages of conventional BCI, improvements based on the use of silicone tubes have become increasingly important [28]. In this study, we introduced a method of MBCI intubation and achieved a good result without more invasive injury using a polyurethane elastomer tube. The MBCI has many advantages. First, when evaluated for invasiveness, the nasal stenting is traditionally considered a more invasive technique because it is usually difficult to clamp the inserted antegrade suture crossing the inferior turbinate out of the inferior meatus, and it often needs to be assisted by an intranasal endoscope [11, 29]. Compared with the traditional BCI, the polyurethane using in this MBCI has some advantages. The main material of MBCI is polyurethane elastomer, which has excellent biocompatibility, the toughness, and rigidity [15]. The hardness of polyurethane elastomer is Shore 74D. This hardness ensures that it is not easy to form sharp corner or break when bending. The hardness also can make the tube fixed in the lacrimal duct without any additional skin suture and antegrade suture. No cases of extrusion have been found in this study. Further research is needed for this. Secondly, it is especially suitable for upper and lower canalicular lacerations. At last, because of the hardness of the polyurethane elastomer, the MBCI can alleviate the tissue tension, which can make the tear edge be anastomosed together and healed accurately.

However, there are also some disadvantages. First, although our study did not find it, the risk of stent prolapse still exists, which need more research. Second, as other intubations, the potential risks of the normal lacrimal punctum splitting and canalicular tearing also exist.

The good outcomes of this study confirm that MBCI was simple and safe in our hands. However, the clinical outcomes should be further investigated by subsequent investigations with a larger sample size.

## Data Availability

The data used to support the findings of this study are included within the article.
